# Automated Inspection of Defects in Optical Fiber Connector End Face Using Novel Morphology Approaches

**DOI:** 10.3390/s18051408

**Published:** 2018-05-03

**Authors:** Shuang Mei, Yudan Wang, Guojun Wen, Yang Hu

**Affiliations:** 1 School of Mechanical Engineering and Electronic Infomation, China University of Geosciences, Wuhan 430074, China; meishuang@hust.edu.cn (S.M.); wangyodan@163.com (Y.W.); 2 Wuhan Second Ship Design and Research Institute, Wuhan 430205, China; huyoung2010@foxmail.com

**Keywords:** defect detection, optical fiber end face, morphology approach, min-max ranking filtering, linear detector

## Abstract

Increasing deployment of optical fiber networks and the need for reliable high bandwidth make the task of inspecting optical fiber connector end faces a crucial process that must not be neglected. Traditional end face inspections are usually performed by manual visual methods, which are low in efficiency and poor in precision for long-term industrial applications. More seriously, the inspection results cannot be quantified for subsequent analysis. Aiming at the characteristics of typical defects in the inspection process for optical fiber end faces, we propose a novel method, “difference of min-max ranking filtering” (DO2MR), for detection of region-based defects, e.g., dirt, oil, contamination, pits, and chips, and a special model, a “linear enhancement inspector” (LEI), for the detection of scratches. The DO2MR is a morphology method that intends to determine whether a pixel belongs to a defective region by comparing the difference of gray values of pixels in the neighborhood around the pixel. The LEI is also a morphology method that is designed to search for scratches at different orientations with a special linear detector. These two approaches can be easily integrated into optical inspection equipment for automatic quality verification. As far as we know, this is the first time that complete defect detection methods for optical fiber end faces are available in the literature. Experimental results demonstrate that the proposed DO2MR and LEI models yield good comprehensive performance with high precision and accepted recall rates, and the image-level detection accuracies reach 96.0 and 89.3%, respectively.

## 1. Introduction

A revolution has been brought to the entire field of communication since the advent of optical fiber, which makes it possible to communicate with high speed and large capacity. Because of the characteristics of low loss, wide transmission bandwidth, small volume and low electromagnetic interference, optical fiber communication has been favored and has been developing very quickly. Optical fiber infrastructures are increasingly popular in government, military, business, industrial applications, and daily life [1]. They have gradually replaced the traditional electrical cable communication as an indispensable medium of information transmission.

**Figure 1:**
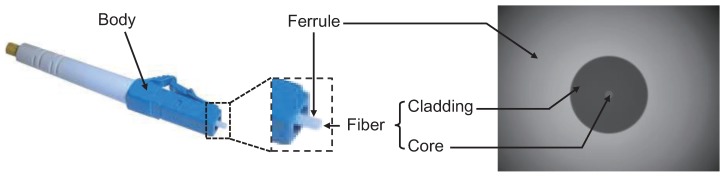
Structure of the LC type optical connector with a magnified view of the ferrule. The right image is collected perpendicular to the end face.

Commonly used optical fiber connector includes four types: ST, SC, FC and LC. They are ordered chronologically since their standardization formulated by the International Electro technical Commission (IEC) [2]. In Figure 1, the structure of the LC type optical connector is presented. It can be seen that the main components of a fiber connector (LC example) include body, ferrule, and fiber parts. The body part houses the ferrule that secures the fiber in place. It also utilizes a latch and key mechanism that aligns the fiber and prevents the rotation of ferrules of two mated connectors [3]. The ferrule part is the center portion of the connector used for the accommodation and exact positioning of a single fiber [2]. The fiber part is the main component of an optical fiber that it is comprised of the cladding and core zones. The cladding zone is a glass layer surrounding the core. It is used to prevent the signal in the core from escaping. The core zone is the critical center layer of the fiber and serves as the conduit that light passes through [3]. When performing fiber-to-fiber mating, various factors may affect the transmission of the signal.

**Figure 2:**
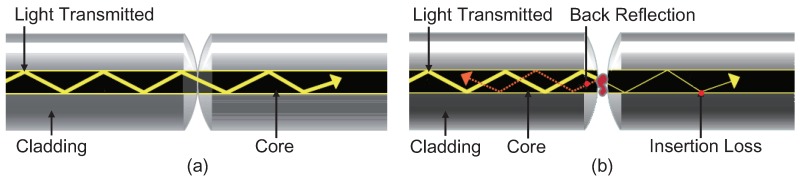
Proper (**a**) and dirty (**b**) fiber connections. Defects in either connector will affect the performance of the mated connection [4].

The presence of dirt, debris, or defects within the fiber core causes not only a loss of signal power (called attenuation or insertion loss) but also a portion of the optical power, which reflects back upstream to the light source (called back reflection), as shown in Figure 2. Both insertion loss and back reflection can have a negative effect on system performance, and defects in either connector will affect the performance of the mated connection [3]. Moreover, they may even permanently damage the connectors and result in irreversible destruction. Therefore, inspecting connector end faces is necessary for providing a reliable, high-performance fiber optic transmission before connecting a new optical fiber into the transmission system.

**Figure 3:**
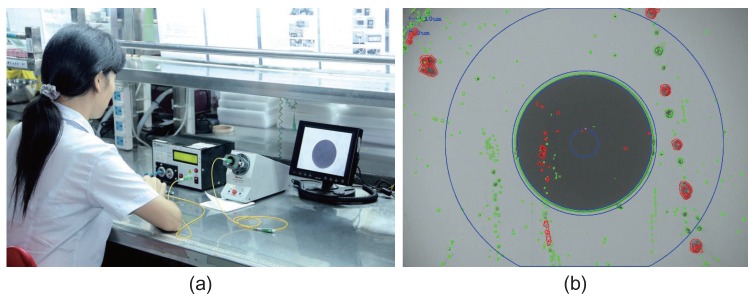
Inspection of optical fiber end faces with (**a**) a human inspector [6] and (**b**) an automated defect detection method.

Traditionally, the inspection of optical fiber end faces is conducted by human inspectors, as shown in Figure 3a. Similar manual visual approaches have many inadequacies, such as poor consistency, non-quantitative evaluation, and high labor costs. In recent years, with the rapid advancement of machine vision and digital image processing techniques, automated defect detection for optical fiber end faces and many other applications [5] has become possible. However, accurate and robust inspection is still a challenging task in this field.

**Figure 4:**
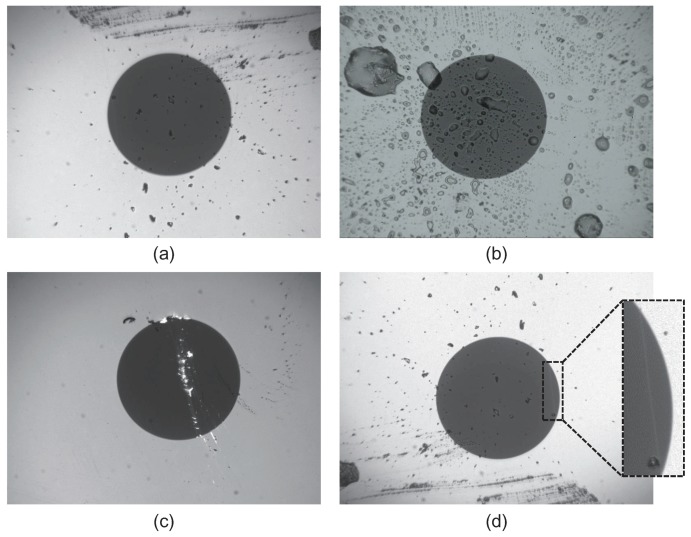
Common types of contamination and defects in optical fiber end faces. From (**a**–**d**): dirt, oil, pit and chip, and scratch types.

Challenges to the automatic defect detection task for optical fiber end faces are mainly embodied in two aspects. First, there are a broader range of different sources that may cause defects of various characteristics in optical fiber end faces, e.g., airborne, hands, clothing, bulkhead adaptor, dusts caps, and test equipment. Sometimes, uneven illumination may affect the inspection performance when conducting the inspection process. As shown in Figure 4, some common types of contamination and defects are presented. They exhibit different properties, and the backgrounds are uneven especially at the corners. Second, the inspection of scratched defects is really difficult and it is a common problem in this field. As shown in Figure 4d, a sample is presented with a magnified view of a scratch. Generally, scratches are prone to be missed during the inspection procedure because of its low contrast characteristic. Moreover, they usually exhibit different orientations and widths, and even cross with each other. All these issues make the inspection of defects in optical fiber end faces a challenging task.

Aiming at the characteristics of typical defects in the inspection process of optical fiber end faces, in this paper, we propose a novel method, “difference of min-max ranking filtering” (DO2MR), for the detection of the region-based defects, e.g., dirt, oil, contamination, pits, and chips, and a special model, a “linear enhancement inspector” (LEI), for the detection of scratches (please note that separately treating these two kinds of defects is necessary according to the latter experimental results, and conforms to industry specifications). The DO2MR is a morphology method that intends to determine whether a pixel belongs to a defective region by comparing the difference between the gray values of the pixels in the neighborhood around the pixel. This method is compatible with region-based defects of various scales, shapes, and structures. The LEI is also a morphology method that is designed to search for scratches at different orientations with a special linear detector. Grayscale difference between scratches with low contrast can be accumulated along the detector direction, so missing scratches inspected by some traditional algorithms may be successfully detected. These two approaches can be easily integrated into optical inspection equipment for automatic quality verification.

The main contributions of this paper can be summarized in the following points.

- As far as we know, this is the first time that complete defect detection algorithms for optical fiber end faces are available in the literature. Many device manufacturers introduce the function but do not illustrate how to implement the detection algorithm, which is the core of the inspection process [7,8]. As for the relevant patents, they are usually very abstract, and only general frameworks are introduced, without specific implementation strategies or algorithm parameters. We also present the quality assessment procedures used in our automatic optical inspection (AOI) equipment, as stated in Section 2.
- The DO2MR and LEI models are aimed at determining the characteristics of typical defects. Experimental results have shown that they have good performance. The average detection accuracies reach 96.0 and 89.3% for region-based defects and scratches, respectively.
- The DO2MR and LEI models can be conducted in a completely unsupervised condition such that no manual intervention is needed. They can be easily utilized in online defect detection lines.

The remainder of this paper is organized as follows. In Section 2, we briefly summarize the automatic quality assessment procedures for optical fiber end faces used in our AOI equipment. Optical fiber connectors are inspected before mating them so as to prevent cross-contamination. In Section 3, the procedures of the proposed DO2MR and LEI models are described in detail. Corresponding inspection performance with these two models is also presented. In Section 4, the “ms-fiber” dataset and the image-level, pixel-level evaluation criteria are introduced. Corresponding quantitative defect detection results for region-based defects and scratches are specified. Implementation details of the proposed models are illustrated in Section 5. Finally, we conclude the paper in Section 6.

## 2. Automatic Quality Assessment for Optical Fiber End Faces

**Figure 5:**
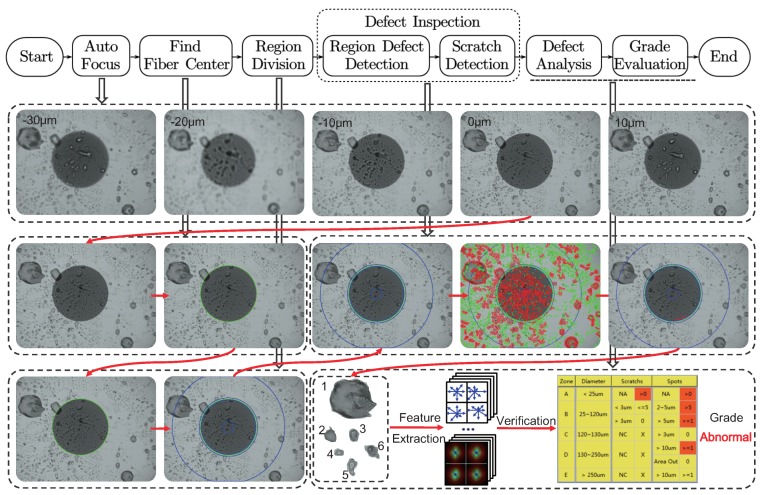
Procedures of automatic quality assessment for optical fiber end faces.

Automatic quality assessment for optical fiber end faces is a complicated process in production lines, and it is necessary to understand complete quality assessment procedures before designing novel defect detection algorithms for optical fiber end faces. As shown in Figure 5, we summarize the steps for quality assessment used in our AOI equipment. Specific introductions are presented as follows.

(1) *AutoFocus* When conducting automatic quality assessment for a candidate optical fiber connector, capturing high-quality digital images is the first important procedure. Due to the errors of manufacturing, distances between end faces of the ferrule and end faces of the body will be slightly different for optical fiber connectors, as shown in Figure 1. They are usually fluctuating within a certain range (−30∼30 μm in our test). This issue leads to a slight difference in the distance between the optical fiber connector end face and the imaging sensor when inserting the connector into a slot for inspecting. However, even this slight difference may cause obvious changes in the image because of the high magnification lens used for inspecting the connector end faces (400× for images in Figure 5). As shown in Figure 5, we present images captured when distances between the connector end face and the imaging sensor deviate from the best focus surface by −30, −20, −10, 0, and 10 μm. It can be seen from these figures that selecting efficient and excellent autofocus algorithm is necessary. Detecting defect accurately is possible only by capturing clear images. Generally, gradient-based autofocus algorithms are widely used to judge the focus state of the camera [9,10]. After capturing high quality images, subsequent defect detection and analysis can be carried out. (2) *Find Fiber Center* Defects in different sections of an optical fiber end face may exhibit different effects for signal transmission. The outer contour of the cladding layer usually serves as the basis for determining the center of the fiber connector, as shown in Figure 5. After determining the center, sections can be segmented. However, the requirement for the algorithm to find the center is very strict, as it must be robust to the interference of dirt and contamination, and it also needs to be compatible with slight defocused conditions. In practical applications, Hough-transform-based methods [11,12] and other specially designed methods [13] are usually applied for circle finding. (3) *Region Division* According to the standard IEC 61300-3-35 (“Fibre Optic Interconnecting Devices and Passive Components—Basic Test and Measurement Procedures”) [14], images of the optical fiber end faces can be divided into zones that are used to prioritize evaluation criteria. For example, the LC type connector can be divided into four zones: the core zone, cladding zone, adhesive zone, and contact zone (blue circles from small to large in Figure 5). Different failure criteria for region-based defects and scratches can be queried for each zone. (4) *Defect Inspection* The defect detection algorithms for region-based defects and scratches are the core of our work, and they will be specified later in this paper. This part makes a quality assessment of optical fiber end faces challenging. (5) *Defect Analysis* As shown in Figure 5, the defect analysis procedure is a process for feature extraction of the segmented defects. Feature extraction is a classical topic in feature engineering that many researchers have devoted to this field [15,16,17]. In this procedure for defects in optical fiber connector end faces, we mainly use handcrafted descriptors for feature representation, e.g., the geometric dimensions, areas, entropy, grayscale moments, and anisotropy. (6) *Grade Evaluation* With the feature representation, the optical fiber connector sample can be graded according to the criteria in the IEC 61300-3-35 standard. As shown in Figure 5, specific issues can be marked and analyzed quantitatively.

## 3. Proposed Methods

In this section, procedures of the proposed morphology approaches for optical fiber end face defect inspection are discussed in detail. They include the detection of general-region-based (e.g., dirt, oil, contamination, pits, and chips) and scratched defects. As the attributes and characteristics of these two kinds of defects are quite different, we will illustrate them separately. Specific descriptions are as follows.

### 3.1. Detection of the Region-Based Defects: Difference of Min-Max Ranking Filter

**Figure 6:**
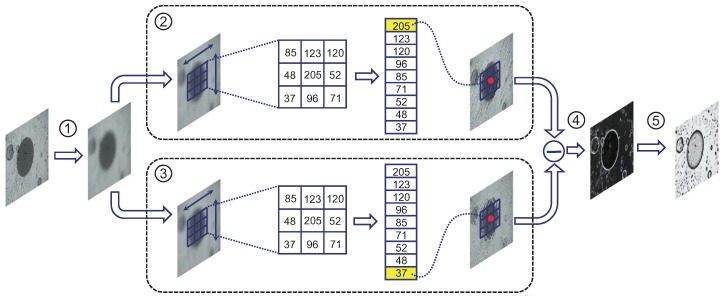
Architecture of the DO2MR filter approach, which is designed mainly for the region-based defects. It includes ① image preprocessing, ② maximum filtering, ③ minimum filtering, ④ residual generation, and ⑤ threshold segmentation steps.

As shown in Figure 6, the architecture of the proposed DO2MR filter approach is illustrated. This approach mainly includes image preprocessing, maximum filtering, minimum filtering, residual generation, and threshold segmentation steps. It is designed mainly for the region-based defects, e.g., dirt, oil, contamination, pits, and chips. As stated before, these defects usually exhibit varied scales, gray values, and shapes. Moreover, they are easily affected by uneven illumination. How the steps of the DO2MR approach are devised to gain inspection robustness will be described.

*Image Preprocessing*: Defect detection is a field with high accuracy. In order to reduce the influence of the image quality on defect detection results, image preprocessing, which is mainly related to the image denoising process, is needed before segmenting defects. In procedures of the optical inspection process, acquisition, transmission, and transformation of a digital image may lead to the introduction of noise, which causes the loss of image details. Here, we use Gauss filtering to smooth the image. This filter uses the weighted gray values of the neighborhood pixels determined by the filter mask to replace the value of each pixel in the image. Given a 1-channel, n-dimensional image $I:\left\{X\subseteq R^{n}\right\}$, the smoothed process is the function $F_{\sigma_{1},\sigma_{2}}:\left\{X\subseteq R^{n}\right\}\to \left\{Z\subseteq R^{n}\right\}$, so the smoothed image $I_{s}$ can be expressed as

(1) $$I_{s}=I*H=I*\frac{1}{2\pi \sigma_{1}\sigma_{2}}\exp\left[-\frac{1}{2}\left(\frac{{\left(x-u\right)}^{2}}{\sigma_{1}^{2}}+\frac{{\left(y-v\right)}^{2}}{\sigma_{2}^{2}}\right)\right]$$

where (u,v) refers to the center of the Gauss kernel, and $\sigma_{1},\sigma_{2}$ denote the standard deviation in the *x* and *y* directions. In the practical operation, the filter template *H* will be discretized, so the smoothing process can be represented as

(2) $$I_{s}=I*\tilde{H}=\sum_{s=-a}^{a}\sum_{t=-b}^{b}I\left(x+s,y+t\right)w\left(s,t\right)$$

where a,b are halves the width and height of the filter template $\tilde{H}$, and $w\left(s,t\right)$ denotes the discrete weight value in $\tilde{H}$ at position $\left(s,t\right)$. After preprocessing, the process will be divided into two streams for the maximum and minimum filtering so as to highlight defects by the contrast difference.

**Figure 7:**
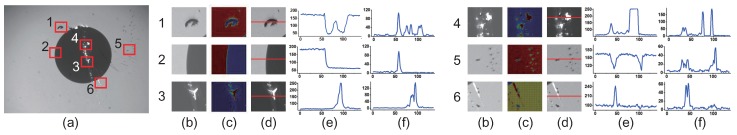
Processes exhibiting the performance of the one-dimensional min-max ranking filtering approach on an optical fiber image. From left to right: (**a**) the raw defective image and (**b**) some randomly selected subregions, (**c**) the corresponding heat maps, (**d**) the sampling position of the one-dimensional data in each subregion, (**e**) the grayscale curve for corresponding one-dimensional data, and (**f**) the corresponding result utilizing the DO2MR filtering approach (best viewed in color).

*Min-Max Ranking Filtering*: The change of gray values in the normal background area is usually relatively smooth, while that in the area corresponding to a defect is usually mutational and defects can be seen as isolated regions in the gentle background. Generally, for the whole image, the influence of a defective target is very slight, but for the local neighborhood, the influence of a defect target on the gray level in the neighborhood may be very obvious. Therefore, methods based on local statistical analysis are more suitable strategies for defect detection. The purpose of the DO2MR approach is to determine whether a pixel belongs to a defective region by comparing the difference of the gray values of pixels in the neighborhood around the pixel. The difference map is obtained from the residual of two images after nonlinear filtering operations, as shown in the ②, ③, and ④ phases in Figure 6. Here we will first illustrate this method with an one-dimensional signal as an example. Supposing the one-dimensional signal is f(t), in order to detect whether there is a burr, mutation, or anomaly in the time domain in f(t), we define a new symbol r(t) to denote the mutation of the signal in time domain. By using DO2MR, r(t) can be expressed as

(3) $$\begin{array}{c} \Delta f\left(t\right)=\min_{t\in \left(t-\Delta t,\;t+\Delta t\right)}f\left(t\right)-\max_{t\in \left(t-\Delta t,\;t+\Delta t\right)}f\left(t\right)\\ r\left(t\right)=d\left(\Delta f\left(t\right)\right) \end{array}$$

where $\Delta f\left(t\right)$ refers to the difference of the signal values in interval $\left(t-\Delta t,\;t+\Delta t\right)$, and $d\left(\cdot \right)$ denotes the metric function. By using |·|, the metric indicator can be expressed as $\left|\Delta f\left(t\right)\right|$. Please note that the expression of $r\left(t\right)$ is quite similar to the partial derivative of the signal $f\left(t\right)$, but they are different. The difference between the maximum and minimum value in the neighborhood in $r\left(t\right)$ is helpful to exhibit weak burrs and defects. Taking the optical fiber connector end face image with pits and chips in Figure 7 as an example, we randomly select six regions of interest (ROIs) and extract a one-dimensional signal (the red line in Figure 7d) in each region. As shown in Figure 7e for these signals, it can be seen that they have different characteristics and therefore have good representativeness. In Figure 7f, residual plots obtained by the min-max ranking method are presented. Regions with mutations or burrs in the original signals are well highlighted, e.g., Signal 1 and 5 in Figure 7f.

**Figure 8:**
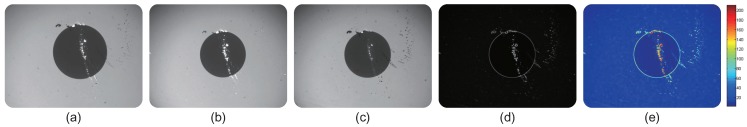
Processes exhibiting performance of the min-max ranking filtering approach on an optical fiber image (two-dimensional). From left to right: (**a**) the raw defective image, (**b**) result of the maximum filtering process, (**c**) result of the minimum filtering process, (**d**) difference of the maximum and minimum filtering processes, and (**e**) the corresponding heat map image (best viewed in color).

As for a two-dimensional signal, the expression is very similar. Assuming that the input two-dimensional signal is $I_{s}$, after the min-max ranking filtering approach, the following intermediate representations can be obtained. Respectively, they are

(4) $$\begin{array}{c} I_{\min}\left(x,y\right)={\left.\min I_{s}\left(x,y\right)\right|}_{x\in \left[x-w/2,x+w/2\right],y\in \left[y-h/2,y+h/2\right]}\\ I_{\max}\left(x,y\right)={\left.\max I_{s}\left(x,y\right)\right|}_{x\in \left[x-w/2,x+w/2\right],y\in \left[y-h/2,y+h/2\right]}\\ I_{r}\left(x,y\right)=I_{\max}\left(x,y\right)-I_{\min}\left(x,y\right) \end{array}$$

where $I_{\min}$ and $I_{\max}$ denote the results after the maximum and minimum filtering processes, $I_{r}$ refers to the corresponding residual map, as shown in Phase ④ in Figure 6. In Figure 8, processes exhibiting performance of the min-max ranking filtering approach on an optical fiber image are presented. From the residual result in Figure 8d and the heat map image in Figure 8e, it can be seen that even tiny defects can also be captured with the proposed DO2MR model.

*Threshold Segmentation*: After obtaining the residual image, a threshold segmentation approach is usually needed to highlight candidate defects. Here, a sigma-based segmentation method is utilized. Prior to its detection, we first smooth the residual image using a 3 × 3 median filter. Let $I_{\hat{r}}\left(x,y\right)$ be the grayscale of the pixel located at the *x* column and the *y* row in the residual image; let *W* and *H* be the width and height, respectively. The grayscale mean (μ) and standard deviation (σ) can be derived as follows:

(5) $$\begin{array}{c} \mu =\frac{1}{W\cdot H}\sum_{x=0}^{W-1}\sum_{y=0}^{H-1}I_{\hat{r}}\left(x,y\right)\\ \sigma =\sqrt{\frac{1}{W\cdot H}\sum_{x=0}^{W-1}\sum_{y=0}^{H-1}{\left(I_{\hat{r}}\left(x,y\right)-\mu \right)}^{2}}. \end{array}$$

Since grayscales at the edges of the defects in the residual image possess slightly higher values than those of their neighboring pixels, we use the following criterion to obtain the resulting image.

(6) $$I_{B}\left(x,y\right)=\left\{\begin{array}{c} \begin{array}{cc} 255 & \mathrm{if}\;I_{\hat{r}}\left(x,y\right)-\mu >\gamma \cdot \sigma \end{array}\\ \begin{array}{cc} 0 & \mathrm{otherwise} \end{array} \end{array}\right.$$

where $I_{B}$ denotes the binary image after segmentation, and γ is a hyper-parameter used to adjust the detection sensitivity. The smaller the γ, the more sensitive it is to the detection of defects, but it may cause some false detections. On the contrary, the greater the γ, the more relaxed the detection condition is, and the risk of missing detection will also exist. In later experiments, the parameter γ is determined by cross-validation. Please note that both the parameter μ and σ are determined based on the defect-free samples. After defect segmentation, the morphological opening process can be conducted to eliminate small and isolated islands. At this point, the processes of defect detection for region-based defects are completed. Then methods of connected domain analysis can be used to describe and analyze these defects for subsequent quality assessment.

### 3.2. Detection of the Scratched Defects: Linear Enhancement Inspection

As stated before, detectionof scratched defects is a challenging task in the field of optical fiber inspection. The main characteristic of these defects is the low contrast visualization, which may cause detection to be missed, even by experienced inspectors. The proposed LEI method is a special approach that is designed primarily for such defects. It mainly includes four processes: image enhancement, scratch searching, scratch segmentation, and result synthesization processes. Detailed descriptions are presented as follows.

*Image Enhancement*: Image enhancement is necessary to modify the dynamic range and contrast of the image, especially for low-contrast scratches. Here, a histogram modeling technique, the histogram equalization, is utilized in this process. This operator employs a monotonic, non-linear mapping, which reassigns the intensity values of pixels in the input image such that the output image contains a uniform distribution of intensities [18]. Consider a discrete grayscale image {x} and let $n_{i}$ be the number of occurrences of gray level *i*. The probability of the occurrence for a pixel of level *i* in the image is

(7) $$p_{x}\left(i\right)=p\left(x=i\right)=\frac{n_{i}}{n},\;\;0\leq i<L$$

where *n* is the total number of pixels in the image, *L* is the total number of gray levels (typical 256), and $p_{x}\left(i\right)$ is in fact the image’s histogram for pixel value *i*, normalized to interval [0, 1] [18]. Let us also define the cumulative distribution function corresponding to $p_{x}$ as

(8) $$T_{x}\left(i\right)=\sum_{i=0}^{j}p_{x}\left(j\right)=\sum_{i=0}^{j}\frac{n_{i}}{n}.$$

Therefore, the new image {y} can be produced according to the transformation below.

(9) $$s_{y}\left(i\right)=\left\lfloor G*T_{x}\left(i\right)-1\right\rfloor ,\;0\leq i<L$$

where *G* is a constant that maps the values into the range of image {y}, and $s_{y}\left(i\right)$ is the transformed value corresponding to the original gray level *i*. After image enhancement, the following scratch highlighting process can be conducted.

*Scratch Searching*: Scratches may exist at any position in an image of the optical fiber end faces, showing any angle. Their characteristics, which are different from other types of defects, are that they are usually slender and have certain linearities. Moreover, the average grayscale value of a scratched region is generally larger than that of the adjacent area in the image because the diffuse reflection will occur when a fiber end face is irradiated (the surface of the scratches region is usually rugged). Therefore, in order to search for scratches, the core goal is to find a way to measure the existence probabilities of scratches at a certain position (x,y) and a certain angle θ. We call this probability the “scratch strength”.

**Figure 9:**
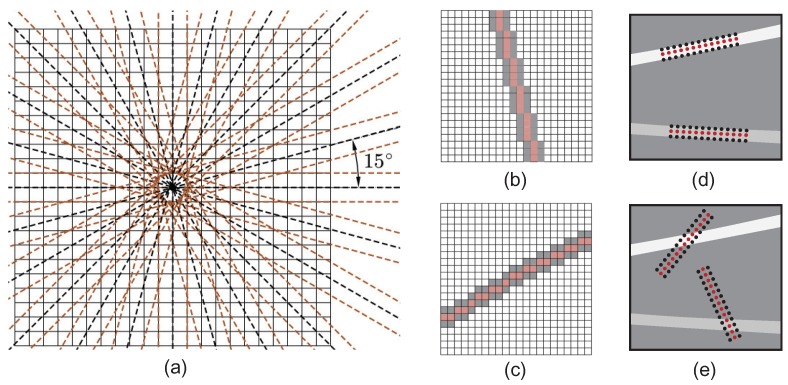
Exhibition of the proposed linear enhancement operator to search for scratches. From left to right: (**a**) 12 searching orientations to evaluate the scratch strength of shaded pixels, linear operators with orientation (**b**) $105^{\circ }$ and (**c**) $30^{\circ }$, and searching situations with (**d**) strong and (**e**) weak scratch strength.

The basic linear detector is illustrated in Figure 9. Scratch strength is evaluated along the lines of fixed length *l* passing through the target pixel (x,y) with different orientations ($15^{\circ }$ of angular resolution in Figure 9a, and this parameter can be adjusted to accommodate specific needs). As shown in Figure 9a, the linear detector is similar to a specially designed filter with two branches, the red and gray ones. We define the difference

(10) $$s_{\theta }\left(x,y\right)=2\cdot f_{\theta }^{r}\left(x,y\right)-f_{\theta }^{g}\left(x,y\right)$$

as the scratch strength at (x,y) with angle θ, where $f_{\theta }^{r}\left(x,y\right)$ and $f_{\theta }^{g}\left(x,y\right)$ denote the average gray level corresponding to the red and gray branches, centered on the pixel (x,y), with angle θ and edge length equal to *l*. Scratch strength is large if the filter is aligned within a scratch; otherwise, the filter is crossed and the scratch strength is lower, as shown in Figure 9d,e. This specially designed detector accumulates the contrast between the pixel in scratches and the surrounding pixels along the filter direction, so it allows us to discriminate scratches from flat background pixels easily. Usually, the length *l* and interval between the red and gray lines in the filter are not fixed. They can be adjusted according to specific needs. In addition, the linear detectors at different orientations are not obtained by interpolation generally, but the pixels to be operated are found by rounding the coordinates of the points on the ideal line [19], as shown in Figure 9b,c (this operation is conducted mainly to reduce the computational complexity).

**Figure 10:**
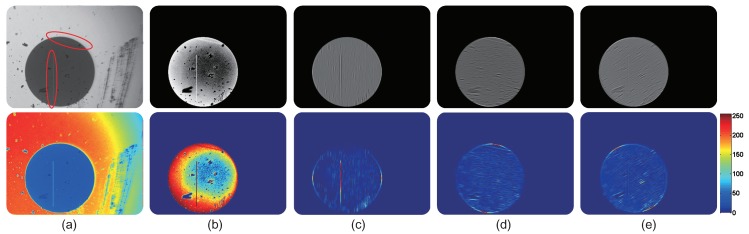
Performance of the scratch searching procedure with the proposed linear enhancement operator. From left to right, in the first row: (**a**) the original defective sample with scratches, (**b**) result after the image enhancement process, and results with the searching strategy at orientation (**c**) $90^{\circ }$, (**d**) $150^{\circ }$ and (**e**) $30^{\circ }$.

The performance of this linear detector on an optical fiber end face image with different orientations is illustrated in Figure 10. This sample image contains two scratches: a real one and a synthetic one. It can be observed that responses are large when orientation of the linear detector is aligned with the corresponding scratches. With these response maps at multiple orientations, scratches can be segmented and synthesized in the following processes.

*Scratch Segmentation*: Scratch segmentation is conducted upon the response maps generated in the last process. Generally, a threshold segmentation approach is needed to highlight the candidate defects. As the strategy used here is similar to the “Threshold Segmentation” approach in Section 3.1, we will not repeat the description. Please note that this process for scratch segmentation is conducted separately for linear detectors with different orientations. After segmentation, results will be synthesized to obtain the final inspection representation.

*Result Synthesization*: Result synthesization is conducted mainly to union scratches in different orientations. Let $\varsigma^{\left(i\cdot \Delta \theta \right)}\left(s\right)$ denote the segmentation binary result at the angle i·Δθ, where Δθ is the searching angle interval. The final scratch detection result can then be expressed as

(11) $$\varsigma \left(x\right)=\varsigma^{\left(\Delta \theta \right)}\left(s\right)|\varsigma^{\left(2\cdot \Delta \theta \right)}\left(s\right)\cdots \left|\varsigma^{\left(i\cdot \Delta \theta \right)}\left(s\right)\cdots \right|\varsigma^{\left(180^{\circ }\right)}\left(s\right)$$

where “|” refers to the OR operation between pixels at the same position. Note that a morphology open operation can be carried out to remove some noise interference if necessary.

Once these processes for scratch detection are complete, defect feature description and analysis can then be carried out.

## 4. Experiments and Discussions

In this section, we will first introduce the datasets and evaluation criteria used in the subsequent experiments. Then, several sets of experiments are presented so as to evaluate the performance of the proposed DO2MR and LEI models. Detailed descriptions are illustrated as follows.

### 4.1. Datasets and Evaluation Criteria

**Figure 11:**
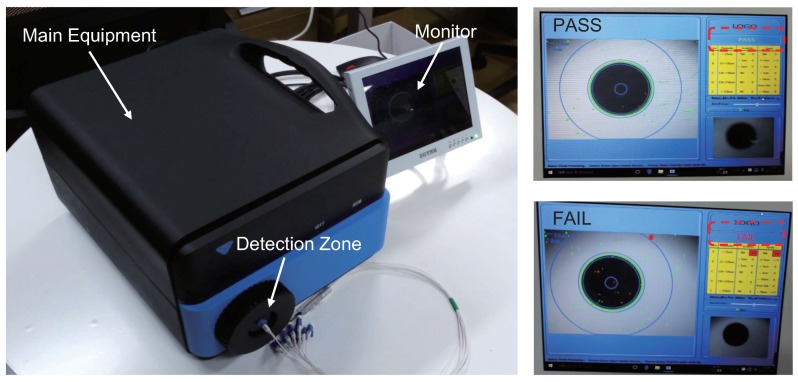
The automatic optical inspection test bench developed for optical fiber end face quality evaluation.

As far as we know, there is no public dataset about optical fiber end face availability for study so far. The samples used in this research were collected with an AOI test bench that we developed and is shown in Figure 11. This test bench is designed mainly to verify the quality of optical fibers quantitatively before they are used in practice. With this equipment, the dataset, which we called “ms-fiber,” was created (https://pan.baidu.com/s/13b3JqBcU1aookvcyOkqxsQ). The dataset includes 116 samples, of which 40 samples are defect-free, 60 are defective with region-based defects, and the remaining 16 are defective with scratches. In later experiments, models will be verified on this dataset.

The evaluation criteria utilized in later experiments include two aspects: the image-level and pixel-level performance metrics. The former measures the accuracy of tagging images that contain defects or are defect-free. It is a widely used metric for defect inspection by measuring detection rates $D_{R}$, false alarm rates $F_{R}$, and detection accuracy $D_{Acc}$. These metrics are defined as follows:

(12) $$\begin{array}{cc} D_{R} & =\frac{TP}{N_{\mathrm{defect}}}\times 100\%\\ F_{R} & =\frac{FP}{N_{\mathrm{defect}\;-\;\mathrm{free}}}\times 100\%\\ D_{Acc} & =\frac{TP+TN}{TP+FN+TN+FP}\times 100\% \end{array}$$

where *TP* and *FN* refer to the numbers of defective samples, which are detected as defective or defect-free. Respectively, *TN* and *FP* refer to the numbers of defect-free samples that are correctly detected as defect-free and falsely detected as defective. Analogously, $N_{\mathrm{defect}-\mathrm{free}}$ and $N_{\mathrm{defect}}$ designate the total numbers of corresponding samples. The pixel-level metric evaluates the inspection accuracy by directly measuring the predicted pixels. Let ${TP}_{p}$ denote the proportion of the correctly segmented defective area in the foreground, the ${FP}_{p}$ is that of the falsely segmented defective area in the background, and ${TN}_{p}$ and ${FN}_{p}$ are similar. The quantitative inspection performance can be evaluated with

(13) $$\begin{array}{cc} Recall & =\frac{{TP}_{p}}{{TP}_{p}+{FN}_{p}}\times 100\%\\ Precision & =\frac{{TP}_{p}}{{TP}_{p}+{FP}_{p}}\times 100\%\\ F1-Measure & =\frac{2\cdot Precision\cdot Recall}{Precision+Recall}\times 100\% \end{array}$$

where the F1−Measure indicator is a comprehensive evaluator upon both the Recall and Precision indicators.

### 4.2. Evaluation of the DO2MR Model (Region-Based Defects)

**Figure 12:**
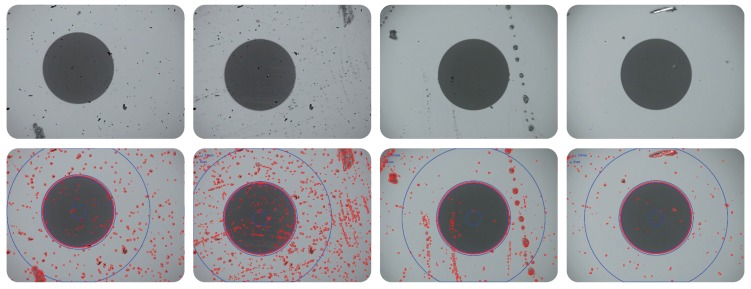
Final defect detection results using the proposed DO2MR method on optical fiber end face images for region-based defects (best viewed in color).

As stated before, the proposed DO2MR model is based on local statistical analysis and its goal is to determine whether a pixel belongs to a defective region by comparing the difference in the gray values of surrounding pixels . As shown in Figure 12, the detection results of some optical fiber end face samples using the proposed DO2MR method are presented. It can be seen that the region-based defects, which exhibit different sizes, shapes, and contrasts, can be accurately inspected. The blue circles in the detection result images refer to different zones of the optical fibers. In these zones, the DO2MR method is compatible.

In order to further verify performance of the proposed DO2MR method, we intend to quantify the detection results by comparing them with the ground truth marked by human inspectors. As far as we know, there is no complete method proposed to inspect defects for optical fiber end faces available so far. Therefore, we will compare the detection performance of DO2MR with some state-of-the-art methods in the automatic surface inspection field (some of them are designed not for the inspection of optical fiber end faces, but their inspection performance is excellent). They are Otsu’s method [20], the sigma-based segmentation (SBS) method [21], the improved-Otsu’s method [22], and the adaptive segmentation method (ASM) [23]. Otsu’s method is a commonly used threshold segmentation strategy. It is applied to automatically perform clustering-based image thresholding, or the reduction of a gray level image to a binary image. The goal of Otsu’s method is searching for the optimum threshold separating the two classes so that their combined spread (intra-class variance) is minimal. In later experiments, Otsu’s method will be separately conducted for the ferrule and cladding zones of the optical fibers because the gray levels of backgrounds in these two regions are different. We call this approach the “Partitioned-Otsu” method. The SBS is an approach designed to reveal low-contrast defects in optical films. It detects defects, such as coatings, streaks, white spots, and foreign particles, and performs well. The improved Otsu is based on 2D information. It segments an image using Otsu criteria and speciallydesigned histogram information. This algorithm avoids the disadvantages of the traditional Otsu method in terms of anti-noise. The ASM segments the product surface from the background and possible defects using a bi-threshold selection method. Experimental results indicate that this algorithm is effective in image segmentation and can satisfy the practical requirements of visual detection of product surface defects.

As shown in Table 1, quantitative image-level defect detection results of the proposed DO2MR model and the compared algorithms on the ms-fiber dataset are presented. It can be seen that the defect detection rate reaches 98.3%, and the total defect inspection accuracy reaches nearly 96.0% using the DO2MR model. In the other four methods, over-detection, mainly caused by uneven illuminations, especially at the corners of the sample images, is very frequent, as shown in Figure 12. This phenomenon directly leads to a high false alarm rate. (The DO2MR is a segmentation method based on local information, while other methods are based on global information. Therefore, these algorithms are more sensitive to illumination changes.) As shown in Table 1, the $F_{R}$ even reaches 55.0% using the SBS method.

Further, as shown in Table 2, quantitative pixel-level defect detection results of the proposed DO2MR model and the compared algorithms on samples with region-based defects are exhibited, and similar conclusions can be drawn. Specifically, the DO2MR model shows a relatively good effect on the Recall indicator, which is utilized to evaluate the detection accuracy. Results on the Precision indicator indicate that the proposed model has good suppression ability for over detection and missing inspection to a certain extent. As for the other four methods, detection performance can be easily affected by the uneven illumination and image noise. Therefore, the Precision performance of these methods is relatively poor, as shown in Table 2. As for the F1−Measure indicator which is a comprehensive evaluator upon the Recall and Precision indicators, the performance is similar. As a whole, the pixel-level defect detection performance of the proposed DO2MR model is better than that of the compared methods. This experiment further demonstrates robustness and effectiveness of the DO2MR model.

### 4.3. Evaluation of the LEI Model (Scratched Defects)

As stated before, scratches are difficult to be detected in optical fiber end faces because of their special characteristics. Generallym they are inspected separately with region-based defects. Whether the proposed LEI model is effective in this task still needs to be validated.

**Figure 13:**
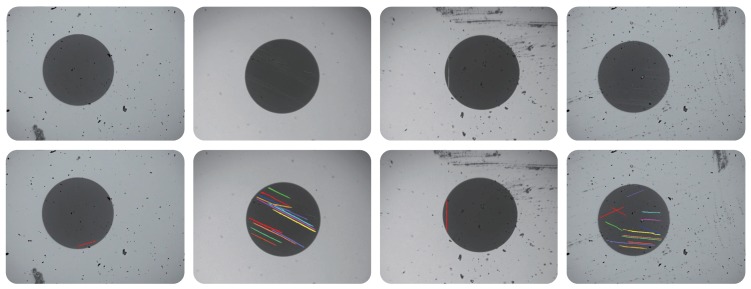
Final defect detection results using the proposed LEI method on optical fiber end face images for scratches (best viewed in color).

As shown in Figure 13, the detection results of some optical fiber end face samples using the proposed LEI model are presented. It can be seen that the scratched defects, which exhibit different lengths, orientations, and contrasts, can be inspected successfully. Adjacent regions with different colors in this figure refer to different scratches (please note that the inspection of scratches is required only in the cladding and core zones of optical fibers).

In order to verify the performance of the proposed LEI model quantitatively, as shown in Table 3 and Table 4, the image-level and pixel-level defect detection results on the defective samples with scratches are presented, respectively. Please note that traditional superior methods are not compared in this experiment because they fail to inspect scratches in two aspects [20,21,23,24]. First, they cannot distinguish scratches from general region-based defects. When scratches occur, the region-based defects will also appear generally. By using these traditional methods, defects can be segmented based on the difference of pixelsǵray values but without further judgement for scratches or region-based defects. Therefore, detecting scratched regions accurately may be a challenge for these methods. Second, they cannot distinguish scratches from each other when multiple scratches occur in an optical fiber end face, as shown in Figure 13. For traditional methods, crossed scratches will be considered as a whole rather than multiple individuals because of the global segmentation mechanism utilized. Here, we compare the proposed LEI model with two specially designed methods, Zana’s [25] and Ricci’s [19] methods, which are also linear morphological operators. Both of these two methods are capable of searching for linear structures of different orientations.

According to Table 3, it can be seen that the proposed LEI model exhibits good comprehensive performance. The total defect inspection accuracy reaches 89.3% and the defect detection rate reaches 87.5%. This experiment shows that the LEI model can well distinguish samples of scratched defects from the normal samples. In addition, results in Table 4 indicate that this model has high segmentation accuracy and good suppression ability for over defection and missing inspection. The Recall reaches 89.4% and the comprehensive evaluator F1−Measure reaches 85.2%. As for the Zana’s and Ricci’s methods, the characteristics of vessel-like patterns are not exactly the same as those of scratches. Specifically, the cross-curvature evaluation strategy in Zana’s method and the orthogonal line filter strategy in Ricci’s method may affect the inspection process for scratches in optical fiber end faces. The quantitative experimental results in these two tables further demonstrate the superiority and effectiveness of the LEI model.

## 5. Implementation Details

The experiments in this paper were conducted on a server with 12 cores with 128 GB of memory. The proposed DO2MR and LEI models were programmed in C++. The computational time of the DO2MR model for an image of 1600 × 1200 pixel for region-based defects was 620 ms on average, and that of the LEI model for scratches was 2240 ms on average, with a searching angle of $15^{\circ }$. The detection methods were verified in an actual inspection line for the LC type optical fibers, and the efficiency met the practical application requirement. Please note that both the DO2MR and LEI are morphology-based methods that can be run in real time on a regular desktop machine).

## 6. Conclusions

In this paper, we propose two novel morphology approaches, the DO2MR and LEI, for the inspection of optical fiber end faces. These two approaches are aimed at determining the characteristics of typical defects in the inspection process. Specifically, the DO2MR is designed for the region-based defects, e.g., dirt, oil, contamination, pits, and chips, and it determines whether a pixel belongs to a defective region by comparing the difference between the gray values of surrounding pixels, while the LEI is designed to search for scratches at different orientations with a special linear detector. These two approaches are morphology-based methods and can be carried out efficiently. As far as we know, this is the first time that complete defect detection algorithms for optical fiber end faces have been made available in the literature. We have also presented the complete quality assessment procedures used in our AOI equipment, which is thus now available as a reference for similar designs. Visual and quantitative results on samples in the ms-fiber dataset have demonstrated effectiveness and robustness of these two models; specifically, the average detection accuracies reach 96.0 and 89.3% for region-based defects and scratches, respectivelyusing these two models. In the future, more experiments will be carried out to further improve the accuracy and stability of the proposed models for more scenarios.

## Figures and Tables

**Table 1:** Average quantitative image-level defect detection results of the proposed DO2MR model and the compared algorithms on the ms-fiber dataset (40 defect-free samples and 60 defective samples with region-based defects).

Criterion (%)	$D_{R}$	$F_{R}$	$D_{\mathrm{Acc}}$
Partitioned-Otsu	85.0 (51/60)	42.5 (17/40)	74.0
SBS	90.0 (54/60)	55.0 (22/40)	72.0
improved-Otsu	88.3 (53/60)	32.5 (13/40)	80.0
ASM	91.6 (55/60)	27.5 (11/40)	84.0
DO2MR	98.3 (59/60)	7.5 (3/40)	96.0

**Table 2:** Average quantitative pixel-level defect detection results of the proposed DO2MR model and the compared algorithms on the ms-fiber dataset (60 defective samples with region-based defects).

Criterion (%)	Recall	Precision	F1−Measure
Partitioned-Otsu	87.4	63.8	73.8
SBS	86.3	57.7	69.2
improved-Otsu	89.2	73.4	80.5
ASM	88.6	79.5	83.8
DO2MR	94.2	88.7	91.4

**Table 3:** Quantitative image-level defect detection results of the proposed LEI model and compared algorithms on the ms-fiber dataset (40 defect-free samples and 16 defective samples with scratches).

Criterion (%)	$D_{R}$	$F_{R}$	$D_{\mathrm{Acc}}$
Zana’s	93.8 (15/16)	42.5 (17/40)	67.9
Ricci’s	75.0 (12/16)	25.0 (10/40)	75.0
LEI	87.5 (14/16)	10.0 (4/40)	89.3

**Table 4:** Average quantitative pixel-level defect detection results of the proposed LEI model and compared algorithms on the ms-fiber dataset (16 defective samples with scratches).

Criterion (%)	Recall	Precision	F1−Measure
Zana’s	86.1	67.5	75.7
Ricci’s	83.8	75.3	79.3
LEI	89.4	81.3	85.2

## Abbreviations

The following abbreviations are used in this manuscript:

AOI	Automatic optical inspection
ASM	Adaptive segmentation method
DO2MR	Difference of min-max ranking filtering
IEC	International electro technical commission
LEI	Linear enhancement inspector
ROI	Region of interest
SBS	Sigma-based segmentation
